# Exploring the lived experience of Long Covid in black and minority ethnic groups in the UK: Protocol for qualitative interviews and art-based methods

**DOI:** 10.1371/journal.pone.0275166

**Published:** 2022-10-03

**Authors:** Nina Smyth, Nisreen A. Alwan, Rebecca Band, Ashish Chaudhry, Carolyn A. Chew-Graham, Dipesh Gopal, Monique Jackson, Tom Kingstone, Alexa Wright, Damien Ridge

**Affiliations:** 1 School of Social Sciences, University of Westminster, London, United Kingdom; 2 School of Primary Care, Population Sciences and Medical Education, Faculty of Medicine, University of Southampton, Southampton, United Kingdom; 3 NIHR Applied Research Collaboration Wessex, Southampton, United Kingdom; 4 NIHR Southampton Biomedical Research Centre, University of Southampton and University Hospital Southampton NHS Foundation Trust, Southampton, United Kingdom; 5 School of Health Sciences, Faculty of Environmental and Life Sciences, University of Southampton, Southampton, United Kingdom; 6 Patient Contributor, General Practitioner & Clinical Lecturer, London, Greater Manchester, United Kingdom; 7 School of Medicine, Faculty of Medicine and Health Sciences, Keele University, Newcastle, Staffordshire, United Kingdom; 8 Wolfson Institute of Population Health, Queen Mary University of London, London, United Kingdom; 9 Patient Contributor, Newcastle, United Kingdom; 10 Midlands Partnership NHS Foundation Trust, Research & Innovation Department, Stafford, United Kingdom; 11 School of Humanities, University of Westminster, London, United Kingdom; Public Library of Science, UNITED STATES

## Abstract

Some people experience prolonged symptoms following an acute COVID-19 infection including fatigue, chest pain and breathlessness, headache and cognitive impairment. When symptoms persist for over 12 weeks following the initial infection, and are not explained by an alternative diagnosis, the term post-COVID-19 syndrome is used, or the patient-defined term of Long Covid. Understanding the lived experiences of Long Covid is crucial to supporting its management. However, research on patient experiences of Long Covid is currently not ethnically diverse enough. The study aim is to explore the lived experience of Long Covid, using qualitative interviews and art-based methods, among people from ethnically diverse backgrounds (in the UK), to better understand wider systems of support and healthcare support needs. Co-created artwork will be used to build on the interview findings. A purposive sampling strategy will be used to gain diverse experiences of Long Covid, sampling by demographics, geographic locations and experiences of Long Covid. Individuals (aged >18 years) from Black and ethnic minority backgrounds, who self-report Long Covid symptoms, will be invited to take part in a semi-structured interview. Interviews will be analysed thematically. A sub-sample of participants will be invited to co-create visual artwork to further explore shared narratives of Long Covid, enhance storytelling and increase understanding about the condition. A patient advisory group, representing diversity in ethnicity and experiences of Long Covid, will inform all research stages. Stakeholder workshops with healthcare professionals and persons, systems or networks important to people’s management of Long Covid, will advise on the integration of findings to inform management of Long Covid. The study will use patient narratives from people from diverse ethnic backgrounds, to raise awareness of Long Covid and help inform management of Long Covid and how wider social systems and networks may inform better healthcare service access and experiences.

## Introduction

Long Covid is the patient-defined term to describe the persistence of symptoms beyond 12 weeks following an acute COVID-19 infection, regardless of the severity of the initial infection, that are not explained by other causes and can last for months or even years [1–3]. The National Institute for Care Excellence (NICE) refer to this phenomenon as post-COVID-19 syndrome [2]. Symptoms tend to be episodic, variable in intensity and recurrent. Symptoms can be physical, cognitive or mental, affecting multiple bodily systems [4, 5]. Symptoms can be debilitating, limiting people’s daily activities, affecting their wellbeing and mental health, their employment and finances [4, 6, 7]. Two million people in the UK (3.1% of the population) currently experience symptoms lasting at least four weeks, and around 1.4 million people experience symptoms lasting for at least 12 weeks [6, 8].

Black and ethnic minority groups have been disproportionality impacted by COVID-19, as evidenced by increased infection and mortality rates [9, 10] hospitalisations [11] and multiorgan dysfunction [12]. However, estimated prevalence rates of Long Covid have not shown themselves to be higher among these populations [6, 13, 14]. This is despite these populations being included in groups most affected by acute COVID-19 infections [13], such as those living in more deprived areas, or having a pre-existing, activity-limiting health condition [6]. With increased prevalence of COVID-19, it is possible that the above groups are at greater risk of suffering increased burden and consequences of Long Covid [15]. Statistics from the Office for National Statistics also suggest that people working in health or social care (professions which include large numbers of ethnic minority communities) are more likely to be affected by Long Covid [16].

Current guidance on the management of Long Covid includes self-management, when investigation to exclude other conditions that might be treatable have been excluded [2, 17]. The scope for self-management can be potentially improved by better understanding the functions of social networks and wider support systems available to individuals, such as the communities in which people are embedded (e.g. e.g. friends, family members, professional), voluntary support and community groups (e.g. faith, religious, ethnic groups), and non-NHS professionals supporting health and wellbeing (e.g. private care, faith/religious leaders, social prescribers, health trainers, spiritualists, herbalists) [18]. For example, increased social interaction with wider support systems promotes self-management, better physical health and greater emotional well-being [19]. Adopting a socially embedded understanding highlights the importance of contexts in which chronic illness can be managed, thus moving us away from individualistic understandings of health and care [20]. People from ethnic minority groups may be more likely to seek alternative forms of support for management of health conditions, such as by prioritising family support, prayer, seeking help from faith leaders and/or complementary and alternative treatments [21, 22]. Whilst these are important for self-management, disparities in support systems and social networks can contribute to inequalities in people’s health [23].

People referred to post-COVID clinics via primary care services are less likely to be from an ethnic minority background [24], and people from ethnic minority backgrounds are less likely to have post-COVID-19 syndrome recorded in their primary care electronic health records [14]. Specialist post-COVID-19 clinics for support from multidisciplinary teams and individualised rehabilitation plans are accessed via primary care services [25]. Minority groups may experience additional barriers to accessing support for their symptoms, such as, lack of trust in professionals, feelings of embarrassment and fear, fatalistic beliefs (shaped by religion/culture) and alternative understandings of causes, symptoms and treatments, lower language proficiency, not presenting to healthcare for certain symptoms and not having culturally-relevant terms to describe medicalised conditions or terms [26–34].

Long Covid is associated with stigma and discrimination, and this is more prevalent in people who have received a clinical diagnosis of Long Covid [35]. Stigma may be perpetuated for people from ethnic minority backgrounds who throughout Western history have reported racial discrimination, a significant cause of health inequalities [36, 37]. For example, discrimination where individuals and institutions, unintentionally, treat minoritised individuals differently e.g. the prioritising but concealing of white systems of knowledge in healthcare by the nature of embeddedness in policies, theories practices and regulations [38]. Cultural racism, linked to negative views and stereotypes attributed to ethnic minority groups, can be consciously or unconsciously adopted and normalised, resulting in the creation and maintenance of structures that perpetuate individual-level biases. This may account for implicit bias observed in healthcare professionals and poorer patient care, treatment experiences and opportunity for medical procedures. Adopting stereotypes as true reflections of communities, can increase psychological distress and result in the adoption of unhealthy behaviours and poor communications with healthcare professionals, and less engagement with healthcare for management of health conditions [37]. Indeed, racial discrimination is a contributory factor of hesitancy towards COVID-19 vaccination [39]. Minority groups are more likely to display distrustful attitudes towards COVID-19 vaccination [40], thus being less likely to take-up COVID-19 vaccinations [20, 41]. COVID-19 vaccination is shown to reduce prevalence of Long Covid [42], thus affected minority groups may potentially be more at risk of developing Long Covid.

Some people with Long Covid report difficulties navigating healthcare access, poor treatment experiences and lack of follow up to care [43–47]. Whilst the lived experience of Long Covid is recognised as crucial to informing Long Covid care and management [2, 48], people from Black and ethnic minority groups are underrepresented in research, meaning important narratives are currently not informing healthcare [49, 50]. Thus, we cannot assume that knowledge informing Long Covid care covers the needs of ethnic minority people. To develop innovative and person-centred care for diverse groups of people living with Long Covid, it is vital to hear from a variety of Black and ethnic minority groups. This approach will work towards ‘de-colonising’ healthcare (i.e. listening to seldom heard voices, being positive and curious about cultural and other differences, and aiming for inclusivity) [51]. Such an approach can help restore empathy and recognition, which has been denied to people living with Long Covid [52]. It may help address issues of equity in healthcare, as well as improve person-centred approaches [51].

### Study aims

Taking a lived experience approach with a ‘decolonising attitude’, the study aims to hear from people from Black and ethnic minority backgrounds, whose perspectives about their illness experiences, healthcare needs and wider support seeking have been largely unheard. Using a qualitative approach and art-based methods, we will explore the lived experience of Long Covid, in people from Black and ethnic minority groups that were disproportionately impacted by COVID-19, including people from Arab, Black, South Asian or mixed backgrounds, regardless of clinical diagnosis status.

## Method

### Design

A qualitative research design using semi-structured interviews with participants who live with Long Covid will be adopted. A sub-sample of participants will co-create artwork to further explore shared narratives of Long Covid, enhance storytelling and increase understanding about the condition. Art-based methods provide an engaging and interactive approach to support exploration of complex understandings and meanings of health, including capturing the social context that might not be easily conveyed via traditional interviews [53]. Such an approach is a novel way to explore deeper understandings of a health condition that may not be easily verbalised or explored in interviews. Artwork increases awareness of health conditions not well understood or managed, better connecting viewers with challenges, and generally highlighting the experiences of people living with health conditions [54].

### Patient and public involvement and engagement

The project received funding from the National Institute for Health and Care Research. Development of the grant involved involvement from people living with Long Covid, as well as healthcare professionals working with Long Covid patients. Two patient co-applicants, from ethnic minority backgrounds living with Long Covid, were named on the funding application and are co-authors of this study protocol (AC, MJ). The scope of the research, study aims, and target sample were discussed with patient members and healthcare professionals of Long Covid support groups (e.g. Long Covid support groups for patients and healthcare professionals; Greater Manchester Long Covid support network) as well as public health and healthcare professionals working in specialist Long Covid National Health Service clinics. A patient advisory group (PAG) has been convened, consisting of seven members, representing diversity in ethnicity, age and Long Covid experiences. Members of the PAG will be involved in all stages of the research, including the design and delivery of study advertisement and interview topics; signposting to networks, organisations and individuals for recruitment of participants and stakeholders; and review and comment on draft themes from the interviews; as well as development of the artwork. They will also be invited to contribute to development of any published outputs and to co-present research at conferences and workshops.

### Study participants

Participants will be people from an ethnic minority background, living in the UK, who currently have Long Covid. Criteria for Long Covid will be in line with NICE [2] and the World Health Organisation Long Covid definitions [3], including ongoing symptoms connected to COVID-19 (probable or confirmed by a test) for at least 12 weeks, not explained by another condition, that generally impact everyday functioning. Eligible participants will be aged over 18 years. Participants will be asked to complete an eligibility form to ascertain age, living location, ethnicity and Long Covid status. Participants whose first language is not English will be invited to take part in the research with an interpreter.

Purposive maximum variation sampling [55] will be used to recruit a diverse range of participants from Arab, Black, South Asian or mixed ethnicity backgrounds. These groups were disproportionality affected by COVID-19 [9, 11, 12], are more likely to live in deprived areas [56], work in healthcare [57, 58], and have pre-existing health conditions [56]. We seek to include participants from a range of demographics (age, gender, subjective social status), geographical locations in the UK, severity of symptoms of Long Covid, including both those diagnosed with Long Covid (who may have accessed healthcare) and those who self-report Long Covid (to ensure we are inclusive of those who ‘fall through the gaps’ of healthcare). Participants will be interviewed who have and have not accessed healthcare for their condition, so as to include a wide range of experiences of people impacted by Long Covid. A multi-stranded recruitment strategy will be used, including, advertisement via traditional media (e.g. radio), social media, support groups, posters in pharmacies, university sites, faith/religious networks and community organisations.

At the end of interviews, participants will be informed about the potential to become involved in artwork-based discussions. Participants interested in this aspect of the research will be sent further information. Of those interested in the artwork, we will select at least six participants to take part. Participants will be selected on their availability to contribute to the artwork-based discussions, and to include a range of socio-demographics and experiences. We aim to include participants representing wide experiences of Long Covid and engagement with healthcare services and other supports. Participants will be offered a £35 shopping voucher for their participation in the interview, and for each artwork-based discussion session.

### Data collection

#### Qualitative interviews

**Fig 1:**
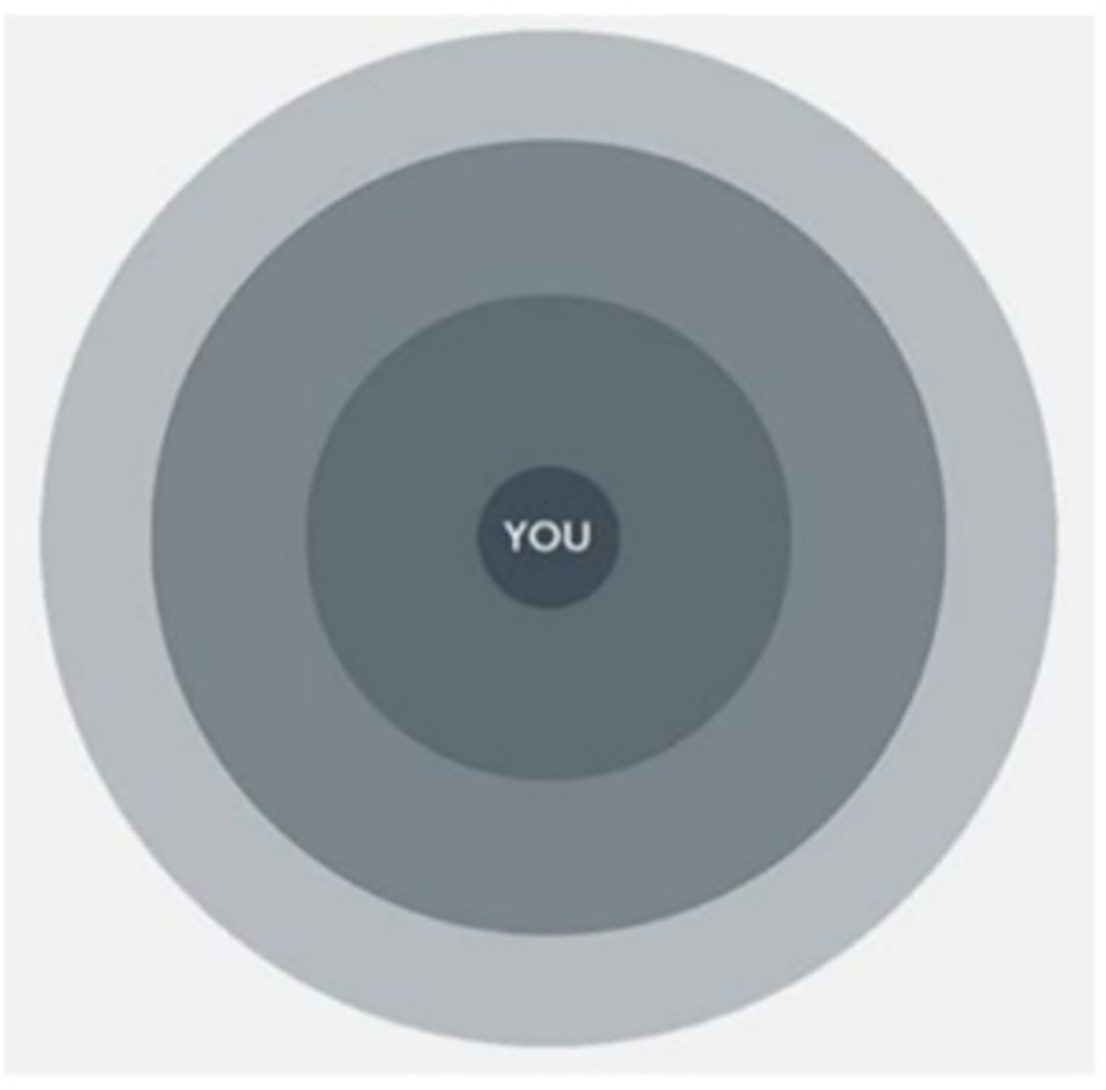
Concentric circles used during interview. The circles nearest the most inner circle represents support systems most important to the participant.

Semi-structured interviews, conducted online, over the telephone or face-to-face, will be guided by an interview schedule, which includes open questions and prompts to explore the nature of symptoms experienced and the impact of symptoms on lives; the support systems (friends, family, community, voluntary, support groups, or alternative and non-healthcare professionals) accessed; what health care services or other supports are called upon; support and treatment preferences and the facilitators/ challenges to accessing appropriate support; recovery of Long Covid; experiences of stigma, discrimination and racism in healthcare; and the role of culture, religion and spirituality in Long Covid. To further enable participants to talk about their support systems, a diagram of concentric circles will be used during the interview (see Fig 1). Participants will be told that the inner circle depicts them and the nearest circle to them represents support systems most important to them in managing their health and everyday life. Whilst moving towards the outer circles which represent support systems decreasingly important to them. Participants are asked to think about who and what support networks or systems they would place in the corresponding circles. This allows participants to focus on the different layers of who and what is important to them in their support network [59]. At the end of interview, participants will also be asked for the date of their COVID-19 infection(s), demographic information (age, gender, ethnicity status, occupation, subjective social status and marital status), geographical location and if they have any other health condition(s). Interview questions will be modified iteratively as data is generated and analysis progresses, suggesting new areas of interest to investigate.

Up to 30 interviews will be conducted, each lasting approximately one hour. Data saturation [60] will inform decisions on when to end data collection, this will be the point at which no new themes are identified in the interview data. Participant recruitment will be monitored to ensure coverage of participants, including across different ethnic minority groups. Interviews will be conducted by several members of the research team including researchers with a mix of ethnicities, qualifications, and experiences: including those with postgraduate qualifications including doctorate; student researchers, and those employed with a research contract or academic status. Three researchers conducting interviews and data analysis are from a White or White other background, and one researcher is from a South Asian background. Experience and training amongst the interview team will vary; with 3 years to over 30 years of qualitative research experience. Practice interviews will be conducted between junior and senior members of the team. Researchers will not interview participants who they have an established relationship with prior to study commencement. Participant’s knowledge of the researcher will be limited to occupation status, demographic details (e.g. gender, and ethnicity) and their interests in the research.

#### Artwork-based discussions

The Artist (AW) will work one-to-one with a sub-sample of participants to explore themes emerging from interviews in more depth using visual media. Themes for discussion will be reviewed at team analysis meetings. During the discussions, imagery, and materials to co-create narratives and imagery of themes, will be discussed. All participants will have one face-to-face meeting with the Artist and follow-up discussions will take place online. The resulting research from these discussions will be reviewed at team analysis meetings. Artworks will represent anonymised personal experiences of Long Covid explored in the interviews. The resulting artwork will be approved by all participants in writing before being exhibited online and in Central London (at University of Westminster central campus). Feedback from viewers of the artwork will be collected on how they connect with the stories depicted in the artwork. The Artist, AW, has extensive experience of translating people’s health experiences into art through participatory research, and frequently works in sensitive areas, such as mental health and chronic health conditions.

### Data analysis

Digitally-recorded interviews will be transcribed verbatim (by a member of the research team or a professional transcriber), with identifying information removed from transcripts. Transcriptions will be checked for accuracy by the interviewer and interested participants will be invited to review the transcript for accuracy and to add additional notes. Interviews that are conducted in a language other than English will include a professional interpreter and interviews will be translated by a professional translator. Professional interpreters, transcribers and translators will sign a confidentiality agreement. Pseudonymised transcripts will be uploaded into NVivo software for coding. A preliminary coding framework will be developed following coding of between 3–5 interviews and finalised as new codes emerge from subsequent interviews. Interview data will be analysed by members of the research team individually and at team group discussions, iteratively using inductive thematic analysis [61], as well as constant comparison, to ensure that all relevant data is compared with similar and contrasting data systematically [62]. Authors involved in data analyses will review all relevant data from interviews. All authors and the patient advisory group will be involved in detailed discussions and debates about codes and themes at team analysis meetings or patient advisory group meetings. Any new understandings from the artwork discussions and artwork exhibition will be used to complement interview data.

Reflexivity will be used throughout collection of data and analysis. The Artist and researchers conducting interviews and data analysis will keep an individual reflective journal. Key issues emerging from individual reflexivity will be discussed at team analysis meetings. The researchers and patient advisory group members represents people from different ethnic backgrounds, people with lived experience of Long Covid and healthcare professionals caring for people with Long Covid.

### Stakeholder workshops

Long Covid patients, public health advisors, general practitioners and non-healthcare professionals (e.g. community figures, faith/religious leaders, occupational health advisors, social prescribing advisors) identified as important to peoples support systems will contribute to stakeholder workshops. The workshops will be focused on understanding how to implement findings into improving better management/care of Long Covid and promote better joined up care and support for people with Long Covid. Three workshops will be held focused on i) discussion of project findings; (ii) public dissemination; and (iii) healthcare dissemination/training.

### Ethics and data management plan

All participants will receive a written debrief about the study, including for the interviews and artwork-based methods. They will provide informed consent either written or electronic for the interviews and artwork-based methods. Consent will confirm they have received sufficient information about the study; they would like to participate in the study; they do not have to answer any question (no questions asked); and they can withdraw at any time (up until data has been analysed). Participants do not have to consent to interviews or art-based discussions being digitally recorded, as the researcher can take notes instead. They also consent to collection, analysis and release of information shared in anonymised form. Participant confidentiality will be maintained at all times unless significant risk to self or others is identified in discussion with the senior research team. Ethical approval for this study has been obtained from the University of Westminster Ethics Committee (ETH2122-1074).

Online forms will be hosted by Qualtrics, and online interviews will be conducted using Microsoft Teams. Data featuring personal details will be securely stored on University of Westminster computers and/or online system. Only pseudonymised data will be shared with investigators/ collaborators. All data will be stored in line with GDPR guidance. Access to participant personal details will be restricted according to researcher role.

## Discussion

The lived experience of Long Covid is crucial to the development and understanding of Long Covid management and care [2, 48]. Long Covid research currently insufficiently considers the experiences of people from Black and ethnic minority backgrounds [49, 50], who are disproportionally affected by COVID-19 [9, 11, 12]. Whilst Long Covid prevalence is not highest for these groups [6], they are likely to face unique consequences of Long Covid, due to existing barriers to seeking healthcare, care not matching their needs, stigma and discrimination within healthcare and by other professionals [26–34, 36–38]. Moreover, these groups are likely to turn to alternative support networks and systems [21, 22], which may facilitate or hinder their access to healthcare services and/or management of Long Covid. Hearing and sharing stories from people from diverse groups can help us to understand how views, experiences and preferences differ, as well as what needs to change to promote quality health care that meets the needs of all people [51].

Diverse patient narratives are crucial to better understanding Long Covid and to inform the design of services for its management and to help inform and educate clinicians treating the condition [2, 48]. This study will use a combination of methods to explore patient experiences, including qualitative interviews and visual art-based methods. Use of visual art-based methods is a creative way of exploring and conveying alternative understandings of Long Covid from the perspective of the person living with Long Covid. As well as exploring different understandings of Long Covid for participants, the artwork produced will be used to increase awareness of Long Covid, in Black and ethnic minority groups, amongst the public and people, systems and networks important to the care of people with Long Covid as well as amongst healthcare professionals. The artwork will represent anonymised individualised experiences of Long Covid, enabling viewers of the artwork to emotionally connect with experiences of illness. The artwork will be showcased via a public engagement event, where people with lived experience of Covid, people who provide both formal and informal care for people with Long Covid, and members of the public will be able to feedback on how they connected with the artwork and how it impacted their understandings and perceptions of Long Covid.

Stakeholder workshops will be conducted to better understand how findings from interviews and art-based methods can best inform better access to health and social care services (e.g. reduce barriers to access and facilitate earlier engagement with appropriate Long Covid services). Minority care experiences will also be informed (e.g. advising on service adaptations and joining up of healthcare services with wider support systems and networks that facilitate self-management of Long Covid).

The findings of the study will be disseminated widely through peer-reviewed publications, scientific conferences, and workshops. In addition, we will aim to disseminate findings using interactive methods, such as training workshop, videos, podcasts, or blogs. All of which will be available to the public on a dedicated website, co-created with people with lived experience of Long Covid. Findings will be shared with different groups, including patients, healthcare professionals, community groups, non-healthcare professionals and commissioners, and policy makers.

### Strengths and limitations of this study

This study will explore the lived experience of Long Covid in individuals among Black and ethnic minority groups whose illness experiences, healthcare needs and wider support seeking in the context of Long Covid remain largely undocumented. A strength of the research is the diversity of the research team and patient advisory groups in terms of ethnicity, disciplinary backgrounds, clinical/non-clinical expertise and lived experience of Long Covid. A variety of methods are used to explore patient narratives of Long Covid, including traditional qualitative interviews and novel creative art-based methods to understand lived experiences and explore social contexts of living with and managing Long Covid. Moreover, art-based methods and stakeholder workshops allows the exploration of experiences or understanding of Long Covid from other perspectives and these methods enable increasing awareness of Long Covid amongst the general public, persons, systems and networks important to management of Long Covid and healthcare professionals/services. A limitation of the study is that participants are self-selecting and may not include individuals who are unable to engage with the research methods and/or individuals who do not identify their symptoms with Long Covid.
