# Supplementary material for: Exploring the lived experience of Long Covid in black and minority ethnic groups in the UK: Protocol for qualitative interviews and art-based methods
Source: PLoS One. 2022 Oct 3;17(10):e0275166. doi: 10.1371/journal.pone.0275166 (PMC9529129; doi:10.1371/journal.pone.0275166)
Supplement: Peer review history

# Editor report: Decision Letter 0

**DOI:** 10.1371/journal.pone.0275166.r001

**Authors:** Avanti Dey

12 Sep 2022

Exploring the lived experience of Long Covid in black and minority ethnic groups in the UK: Protocol for qualitative interviews and art-based methods

PONE-D-22-22503

Dear Dr. Smyth,

We’re pleased to inform you that your manuscript has been judged scientifically suitable for publication and will be formally accepted for publication once it meets all outstanding technical requirements.

Within one week, you’ll receive an e-mail detailing the required amendments. When these have been addressed, you’ll receive a formal acceptance letter and your manuscript will be scheduled for publication.

An invoice for payment will follow shortly after the formal acceptance. To ensure an efficient process, please log into Editorial Manager at http://www.editorialmanager.com/pone/, click the 'Update My Information' link at the top of the page, and double check that your user information is up-to-date. If you have any billing related questions, please contact our Author Billing department directly at authorbilling@plos.org.

If your institution or institutions have a press office, please notify them about your upcoming paper to help maximize its impact. If they’ll be preparing press materials, please inform our press team as soon as possible -- no later than 48 hours after receiving the formal acceptance. Your manuscript will remain under strict press embargo until 2 pm Eastern Time on the date of publication. For more information, please contact onepress@plos.org.

Kind regards,

Avanti Dey, PhD

Staff Editor

PLOS ONE

Journal Requirement:

1. Thank you for stating the following in the Competing Interests section: 

"I have read the journal's policy and the

authors of this manuscript have the following

competing interests:

NAA is co-investigator on NIHR–funded STIMULATE ICP study and has contributed in an advisory capacity to the World Health Organisation and the European Union Commission's Expert Panel on effective ways of investing in health meetings in relation to post-COVID-19 condition. CCG is in receipt of NIHR School for Primary Care Research funding (Symptom patterns and life with post-acute COVID-19 in children and young people: SPLaT-19 cohort and qualitative study). DR is a co-investigator on the Roche funded study, Portraits of Care."

Please confirm that this does not alter your adherence to all PLOS ONE policies on sharing data and materials, by including the following statement: "This does not alter our adherence to  PLOS ONE policies on sharing data and materials.” (as detailed online in our guide for authors http://journals.plos.org/plosone/s/competing-interests). If there are restrictions on sharing of data and/or materials, please state these. Please note that we cannot proceed with consideration of your article until this information has been declared. 

Please respond by return email with your amended Competing Interests Statement and we will change the online submission form on your behalf.

Additional Editor Comments:

We are pleased to inform you that your Study Protocol has been evaluated to be eligible for expedited review and acceptance. Thank you for submitting your work to PLOS ONE.

Reviewers' comments:

# Editor report: Acceptance letter

**DOI:** 10.1371/journal.pone.0275166.r002

**Authors:** Avanti Dey

23 Sep 2022

PONE-D-22-22503 

Exploring the lived experience of Long Covid in black and minority ethnic groups in the UK: Protocol for qualitative interviews and art-based methods 

Dear Dr. Smyth:

I'm pleased to inform you that your manuscript has been deemed suitable for publication in PLOS ONE. Congratulations! Your manuscript is now with our production department. 

If your institution or institutions have a press office, please let them know about your upcoming paper now to help maximize its impact. If they'll be preparing press materials, please inform our press team within the next 48 hours. Your manuscript will remain under strict press embargo until 2 pm Eastern Time on the date of publication. For more information please contact onepress@plos.org.

If we can help with anything else, please email us at plosone@plos.org. 

Thank you for submitting your work to PLOS ONE and supporting open access. 

Kind regards, 

PLOS ONE Editorial Office Staff

on behalf of

Dr. Avanti Dey 

Staff Editor

PLOS ONE
